# Antifibrotic drugs as therapeutic tools in resistant melanoma

**DOI:** 10.15252/emmm.202115449

**Published:** 2022-02-14

**Authors:** Berta Sanchez‐Laorden, M Angela Nieto

**Affiliations:** ^1^ Instituto de Neurociencias (CSIC‐UMH) Sant Joan d’Alacant Spain

**Keywords:** Cancer, Skin

## Abstract

Melanoma is the most aggressive form of skin cancer. Together with the recent advances in immunotherapy, targeted therapy with inhibitors of the Mitogen Activated Protein Kinase (MAPKi) pathway including BRAF and MEK inhibitors has greatly improved the clinical outcome of these patients. Unfortunately, due to genetic and non‐genetic events, many patients develop resistance to MAPKi. Melanoma phenotypic plasticity, understood as the ability of melanoma cells to dynamically transition between different states with varying levels of differentiation/dedifferentiation, is key for melanoma progression. Lineage plasticity has also emerged as an important mechanism of non‐genetic adaptive melanoma drug resistance in the clinic (Arozarena & Wellbrock, 2019), highlighting the need for a deeper characterization of the mechanisms that control this process. In this issue of EMBO Molecular Medicine, Diazzi *et al* (2022) identify a mechanism regulating MAPKi‐induced phenotypic plasticity and resistance, providing evidence to support the use of an anti‐fibrotic drug as a potential novel combinatorial therapeutic approach.

## Graphical abstract

B. Sanchez‐Laorden & A. Nieto discuss the study by Diazzi *et al*, in this issue of *EMBO Mol Med*, that shows that blockade of pro‐fibrotic response prevents targeted therapy‐induced phenotypic plasticity, and thus delays resistance in melanoma.

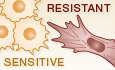

The melanoma “phenotype‐switch” model describes how cells transition in response to microenvironmental signals and Melanocyte Inducing Transcription Factor (MITF) activity from a proliferative/differentiated to a mesenchymal‐like invasive/dedifferentiated state (Hoek & Goding, 2010). Interestingly, phenotypic plasticity is also important to drive drug resistance in melanoma, with the mesenchymal‐like invasive phenotype being associated with MAPKi resistance. Previous work from the authors described that these mesenchymal‐like and drug‐resistant melanoma cells can also acquire myofibroblastic traits to promote a profibrotic response, including collagen deposition and matrix remodelling (Girard *et al*, 2020). In the current study, they extend their investigation to the mechanisms behind this phenotypic switch and the contribution of the associated profibrotic response to drug resistance.

Nintedanib, an anti‐fibrotic drug, has been successfully used to treat patients with idiopathic pulmonary fibrosis (Bonella *et al*, 2015), which prompted Diazzi *et al* to test its impact in preclinical melanoma models. They observed a significant delay in acquired resistance to MAPKi, and an increase in mice survival, associated with a reduction in the mesenchymal and myofibroblastic gene signatures in the tumours. To elucidate the underlying molecular determinants, Diazzi *et al* focused on a set of microRNAs, known as FibroMiRs, previously associated with organ fibrosis. Through an expression screening, they identified mirR‐143‐3p and miR145‐5p as the top candidates involved in melanoma drug‐induced phenotypic switches. As such, drug‐resistant melanoma cells showed increased levels of the mirR‐143/145 cluster. Importantly, MAPK inhibition‐mediated upregulation of the cluster was blocked by Nintedanib, indicating that its antifibrotic activity and the attenuation of melanoma drug resistance are at least in part mediated by preventing the miRs upregulation. Compatible with this, mirR‐143/145 targeting improved the responses to MAPKi, all together highlighting the role of these miRs in the mechanism behind targeted therapy resistance.

FSCN1, a protein involved in the reorganization of the actin cytoskeleton, was identified as an important target of the miR‐143/‐145 cluster in mesenchymal‐like melanoma cells. The authors confirmed the contribution of the miR‐143‐/145 cluster/FSCN1 axis to cytoskeleton rearrangements concomitant with YAP and MRTFA nuclear traslocation and activity, which they had previously associated with MAPKi resistance (Girard *et al*, 2020). Thus, Diazzi *et al* show not only that the MAPKi‐induced expression of the FibromiR‐143‐/145 cluster drives a phenotypic switch towards a mesenchymal‐like phenotype in melanoma but also that it is involved in the acquisition of MAPKi resistance by regulating mechanosensing pathways associated with profibrotic properties.

The differentiated/proliferative to mesenchymal/invasive phenotypic switch in melanoma is accompanied by another switch that involves the expression of the so‐called epithelial‐to‐mesenchymal transition‐inducing transcription factors (EMT‐TFs), and that may be regulated by TGFβ (Pedri *et al*, 2021). EMT is a prominent phenotypic plasticity programme activated both in embryonic development and disease, the latter including cancer and fibrosis (reviewed in Nieto *et al*, 2016). In cancer, EMT is associated with dedifferentiation and invasive potential and, in fibrosis, with dedifferentiation and conversion of fibroblasts into myofibroblasts. EMT has also been associated with therapy resistance in different cancer types (Nieto *et al*, 2016), and evidence of dedifferentiation and drug resistance has been found in melanoma (Arozarena & Wellbrock, 2019), including the co‐existence of neural crest stem cell (NCSC)‐like cells and drug‐tolerant mesenchymal‐like cells (Rambow *et al*, 2018). Thus, both the phenotypic switch and acquired MAPKi resistance are compatible with the activation of an EMT programme (Pedri *et al*, 2021). Interestingly, Diazzi *et al* (2022) show that TGFβ, the most potent inducer of EMT in cancer and fibrosis, activates miR‐143‐/145 cluster expression in melanoma cells and that TGFβ can also activate MRTFA expression in non‐small lung cancer cells (Du *et al*, 2015), linking these two central players now described to lead to MAPKi resistance and fibrosis. In renal fibrosis, EMT is also activated, and reciprocal loops are established between the epithelial cells and the fibrotic microenvironment involving TFGβ signalling. Importantly, dedifferentiation, collagen deposition and inflammation can be reversed by inhibiting EMT (Grande *et al*, 2015). Similarly, Nintedanib reduces TFGβ‐induced miR‐143‐/145 cluster upregulation, and it is likely that its efficacy on MAPKi resistance amelioration is associated with blocking an EMT‐like phenotypic switch.

The genetic programmes activated in drug‐resistant mesenchymal‐like melanoma cells share common features with EMT, including increased TGFβ signalling and high levels of AXL (Rambow *et al*, 2018; Arozarena & Wellbrock, 2019; Pedri *et al*, 2021). Interestingly, EMT‐induced resistance to targeted therapy drives AXL upregulation in non‐small cell lung cancer mesenchymal cells (Nieto *et al*, 2016). Diazzi *et al* show that upregulation of miR‐143‐/145 cluster is associated with increased AXL expression, suggesting that EMT inducers are likely to be involved in miR‐143‐/145 cluster‐driven MAPKi resistance, which remains to be experimentally tested. In summary, evidence points to a contribution of EMT‐like programmes to MAPKi‐driven phenotypic plasticity in melanoma. However, it remains to be elucidated whether EMT transcription factors play a role in melanoma resistance to therapy *in vivo*. Nevertheless, it is clear that targeted therapy with MAPKi in combination with approved anti‐fibrotic drugs could be a step forward in the clinic for melanoma patients and potentially other cancer patients. Finally, TGFβ‐induced MRTFA, together with NFκB/p65, binds to *PD‐L1* promoter, activating its expression and promoting immune escape (9), suggesting that antifibrotic drugs could reinforce responses to immunotherapies and opening further avenues in multiple combination therapies (Fig 1).

**Figure 1:**
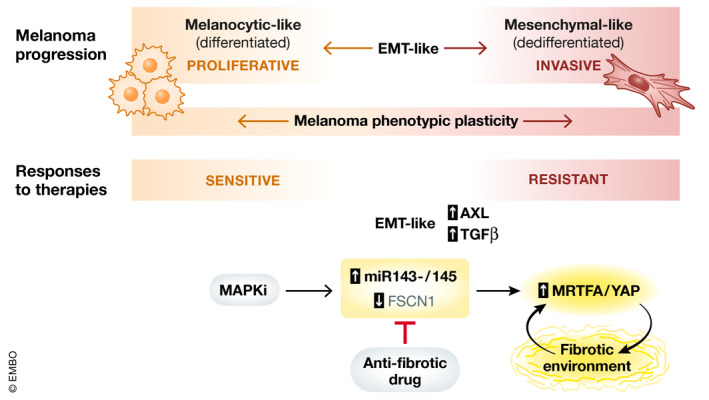
Targeted therapy‐induced melanoma phenotypic plasticity and drug resistance regulation MAPKi induces miR143‐/145 cluster upregulation and promotes the acquisition of drug resistance and fibrosis driven by MRTFA/YAP. This can be ameliorated by the anti‐fibrotic drug Nintedanib. The acquisition of resistance is accompanied by a switch towards a mesenchymal‐like phenotype, suggestive of an EMT‐like process and compatible with that described during melanoma progression. As such, MAPKi resistance in melanoma coincides with an increase in the activity of EMT regulators TGFβ and AXL, which has also been associated with chemoresistance in carcinomas. As the delay in MAPKi resistance induced by Nintedanib is in part mediated by preventing the upregulation of miR143‐/145 expression by TGFβ, this likely concurs with the amelioration of the EMT‐like programme.
